# Periostin, a signal transduction intermediate in TGF-β-induced EMT in U-87MG human glioblastoma cells, and its inhibition by anthocyanidins

**DOI:** 10.18632/oncotarget.25153

**Published:** 2018-04-24

**Authors:** Amira Ouanouki, Sylvie Lamy, Borhane Annabi

**Affiliations:** ^1^ Laboratoire d’Oncologie Moléculaire, Centre de Recherche BioMed, Université du Québec à Montréal, C.P. 8888, Succ. Centre-ville, Montréal, Québec, Canada H3C 3P8

**Keywords:** anthocyanidins, EMT, glioblastoma, periostin, TGF-β

## Abstract

Periostin is a secreted protein that is highly expressed in glioblastoma cells as compared to normal brain tissue, and is therefore considered as a potential biomarker in therapeutic modalities. Its contribution in the cancer cells invasive phenotype is, however, poorly understood. This work investigates the role of periostin in U-87 MG glioblastoma cell invasion, cell migration and in Transforming Growth Factor β (TGF-β)-induced epithelial-mesenchymal transition (EMT). Periostin gene silencing, using small interfering RNA, decreased TGF-β-induced mesenchymal marker expression of fibronectin and vimentin, partly through reduced Smad2, Akt and Fak phosphorylation as well as U-87 MG cell invasion and migration. The effects of anthocyanidins, the most abundant diet-derived flavonoids, were examined on periostin-mediated downstream signaling pathways. Anthocyanidins were found to decrease periostin expression whether added under pre-, co- or post-treatment conditions along with TGF-β, and altered the Akt and Fak signaling pathways. These effects were similar to Galunisertib (LY2157299), a small molecule inhibitor of the TGF-β receptor I kinase. Taken together, our data demonstrate that periostin acts as a central element in TGF-β-induced EMT, which can be prevented by diet-derived anthocyanidins.

## INTRODUCTION

Periostin, originally named osteoblast-specific factor-2 (Osf2), is a 93.3 kDa matricellular protein known to play roles in osteology and tissue repair in the cardiovascular and respiratory systems [1]. It is also involved in tumor development where it promotes cancer cell survival, invasion, migration and angiogenesis leading to epithelial-mesenchymal transition (EMT) and metastasis [2]. Periostin is found in normal adult tissues such as aorta, stomach, breast, lung, thyroid, colon, ovary and prostate, whereas it is overexpressed in various cancer types including neuroblastoma, head and neck, colon, thyroid, ovarian and breast cancers [3]. Studies suggest that the molecular processes involved in metastasis, invasion and cell survival may be regulated through periostin functions [4]. It is believed that periostin plays a dual role in this respect as it can also act as a suppressor of tumor progression in several types of cancer [2].

EMT plays an important role in physiological processes such as in embryonic development, but also in pathologic processes such as tumor progression [5]. EMT promotes dissemination of primary epithelial cancer cells to distant sites thus regulating the metastasis and malignant behavior of the primary tumor [6]. EMT is triggered by several growth factors, including TGF-β, and is characterized by the induction of mesenchymal markers such as periostin, fibronectin, β-catenin and vimentin and by the loss of epithelial markers such as occludin, claudin and E-cadherin [7]. Several studies have suggested that periostin expression and its secretion, driven by TGF-β, play a role as an EMT inducer [8].

Secreted periostin acts, in part, through integrin receptors αvβ3 and αvβ5 to activate the Akt survival and Fak invasive pathways leading to EMT and metastasis [9, 10]. On the other hand, little is known about the role for periostin in glioma, aside from its expression being correlated with glioma tumor grade and survival [9]. The mechanisms by which periostin contributes to glioma malignancy therefore requires more investigation.

Glioblastoma multiforme (GBM) is the most common malignant tumor of the primary central nervous system (CNS) [11]. GBM is highly invasive which makes it resistant to radiotherapy and chemotherapy, and so the median survival time of GBM patients is less than 15 months [12]. Current GBM treatment includes maximal resection followed by radiotherapy along with temozolomide as chemotherapy. Despite this therapeutic regimen, patients suffer recurrence and only 3–5% survive 5 years after diagnosis [13]. Given that several EMT-targeting molecules have advanced into clinical trials, with chemical structures often inspired from those of natural diet-derived compounds, assessment of their chemopreventive properties may lead to novel strategy in metastasis suppression [14].

In the present study, we tested anthocyanidins, a class of flavonoids with a wide range of reported health-promoting pharmacological effects on obesity control, cancer and cardiovascular disease [15]. Specifically, we investigated the role of periostin in glioblastoma cell invasion, cell migration and on TGF-β-induced EMT. We also assessed the effects of anthocyanidins on signaling pathways and compared them to those of Galunisertib, a phase II experimental drug, known to also target TGF-β receptor-mediated signaling in GBM [16, 17].

## RESULTS

### TGF-β induces periostin expression in U-87 MG Cells

**Figure 1:**
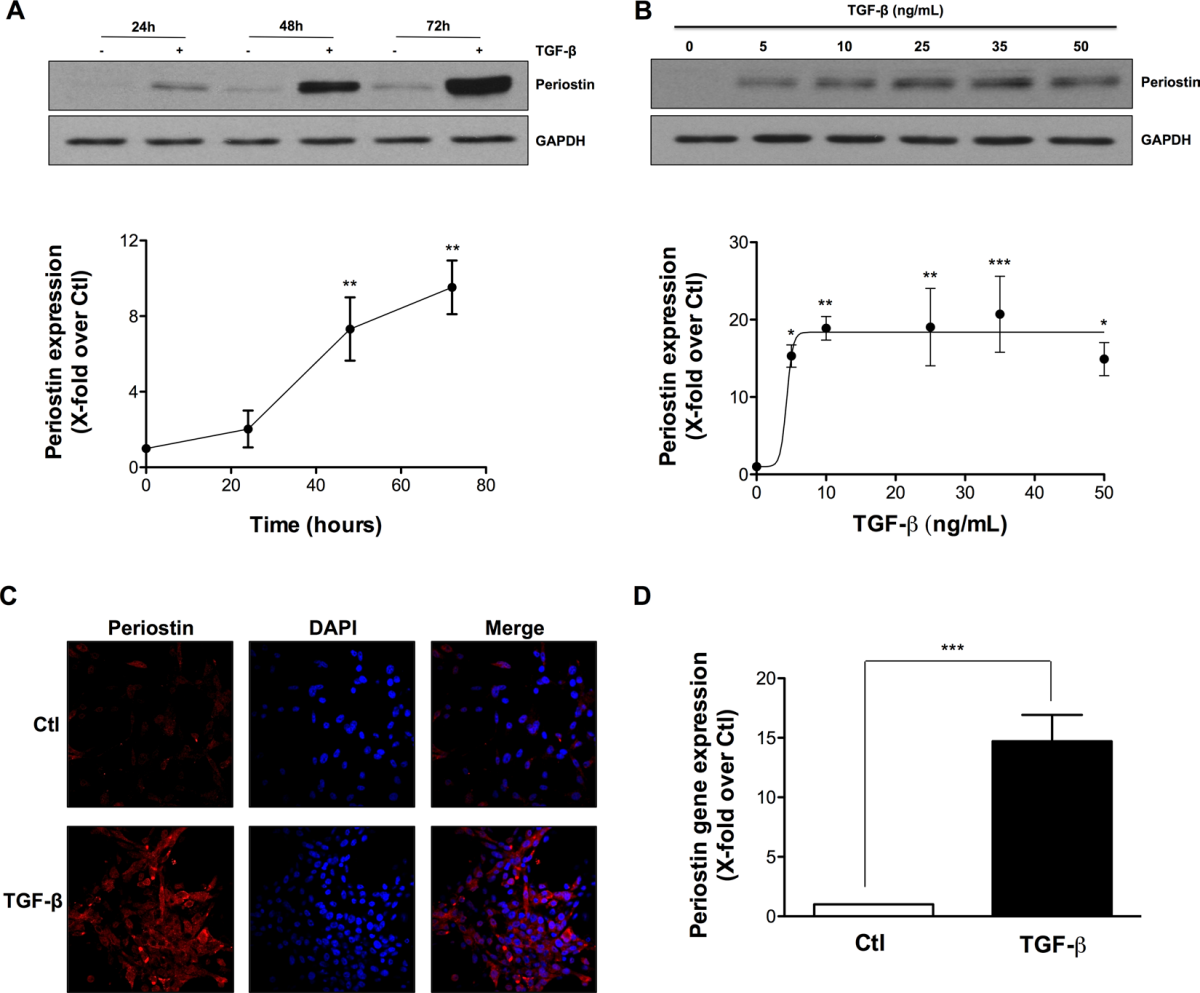
TGF-β induces periostin expression in U-87 MG Cells U-87 MG cells were exposed to TGF-β. Western blot analysis demonstrated levels of periostin protein expression after (**A**) treatment with 10 ng/mL TGF-β for 24, 48 and 72 h or (**B**) treatment with different concentrations of TGF-β for 48 h. Densitometric analysis is representative of three or more independent experiments. (**C**) Photomicrographs show the immunostaining of periostin (red) and nuclei (blue) using fluorescence microscopy. (**D**) The effect of TGF-β on periostin gene expression was evaluated by real-time qPCR. Statistically significant differences were calculated by unpaired Student’s *t* test (D) and one-way ANOVA followed by Bonferroni’s test (A, B) (^*^P < 0.05, ^**^P < 0.01, and ^***^P < 0.001 versus control cells).

We measured the effects of TGF-β on periostin expression in U-87 MG cells and found that it induced the expression of periostin in a time-dependant manner (Figure 1A) to reach a plateau at 10 ng/mL TGF-β (Figure 1B). TGF-β-induced periostin expression was also confirmed by immunofluorescence (Figure 1C). Periostin gene expression levels were also found up-regulated upon TGF-β treatment (Figure 1D). Taken together, these results demonstrate that periostin is efficiently regulated upon TGF-β-mediated signaling in U-87 MG cells.

### Periostin repression alters TGF-β-induced EMT biomarkers expression

**Figure 2:**
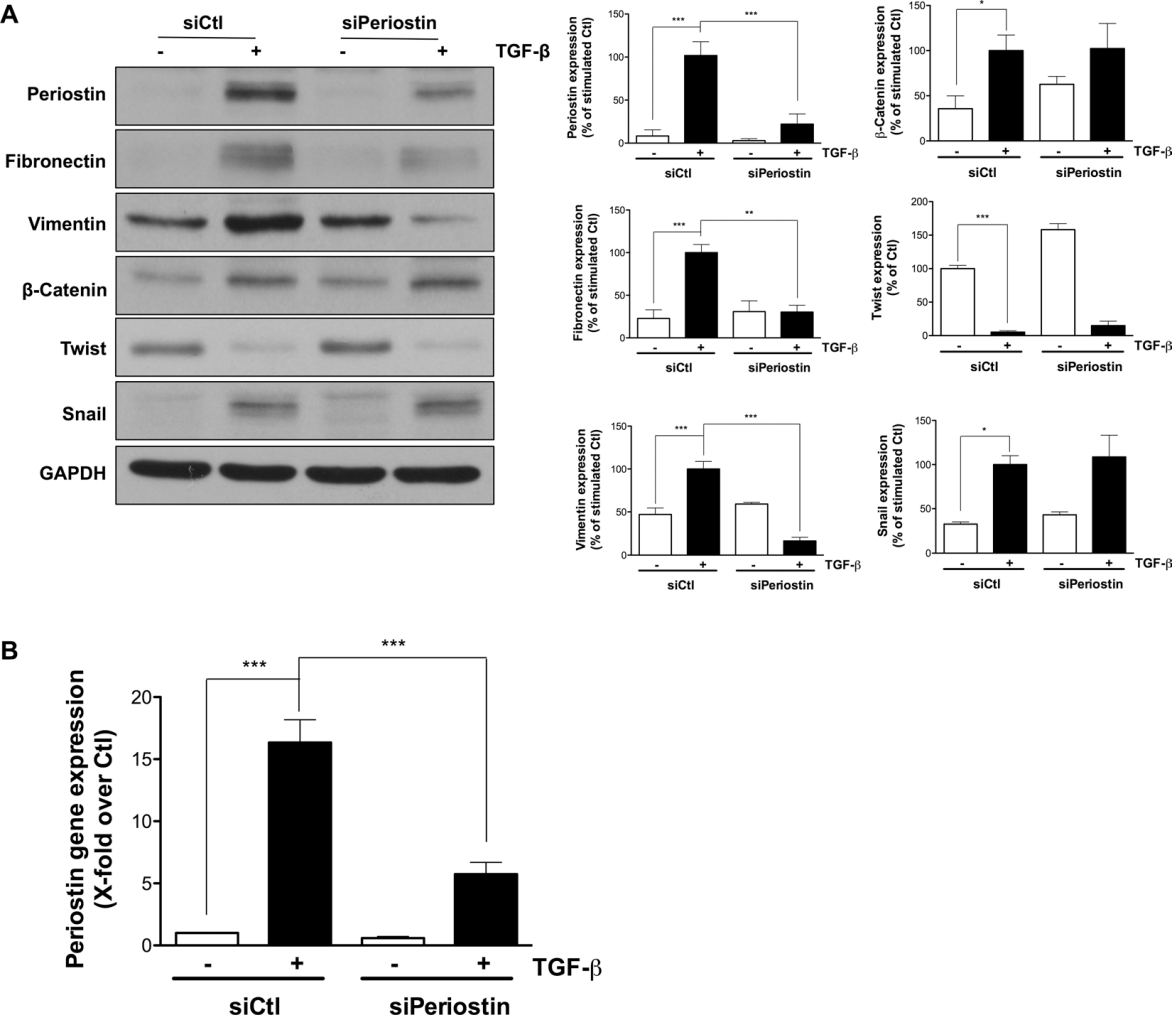
Periostin repression alters TGF-β-induced EMT biomarkers expression U-87 MG cells were transfected with 2 nM periostin siRNA for 24 h. (**A**) Western blot analysis shows protein expression levels of EMT markers. Densitometric analysis is representative of three or more independent experiments. (**B**) RT-qPCR quantification of periostin gene expression following treatment with 10 ng/mL TGF-β for 48 h. Statistically significant differences were calculated by one-way ANOVA followed by Bonferroni’s test (^*^P < 0.05, ^**^P < 0.01, and ^***^P < 0.001 versus control cells).

Next, the role of periostin in TGF-β-induced EMT was evaluated. Periostin was silenced using siRNA, TGF-β was added to cells for 48 h, and the protein expression of several EMT markers evaluated by immunoblotting. Periostin repression was found to reduce TGF-β-induced expression of both fibronectin and vimentin by 70 and 80%, respectively. β-Catenin, twist and Snail expression were unaffected (Figure 2A). Periostin gene silencing was confirmed by quantitative PCR in the presence of TGF-β (Figure 2B). These results position periostin as a new potential signal transduction intermediate within the TGF-β-induced EMT process.

### Pre-, co- and post-TGF-β treatments with anthocyanidins affect periostin expression

**Figure 3:**
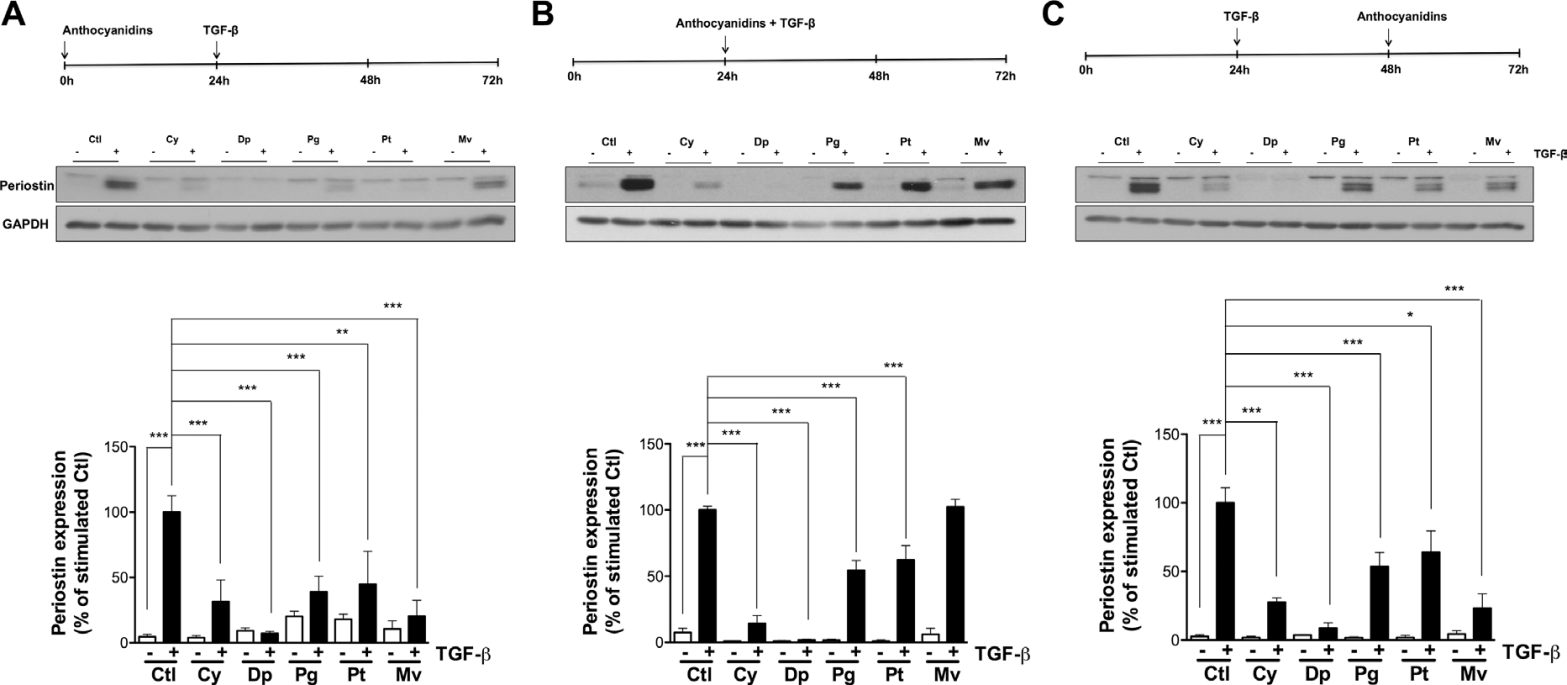
Pre-, co- and post-TGF-β treatment with anthocyanidins effects on periostin expression U-87 MG cells were incubated in serum-free medium containing (or lacking) 50 mM of the indicated anthocyanidin (Cy, cyanidin; Dp, delphinidin; Pg, pelargonidin; Pt, petunidin; Mv, malvidin). Cells were (**A**) pre-treated with anthocyanidins for 24 h, followed by 10 ng/mL TGF-β for 48 h, or (**B**) serum-starved for 24 h and co-treated with anthocyanidins and TGF-β for 48 h, or (**C**) serum-starved for 24 h followed by the addition of TGF-β for 48 h and post-treated with anthocyanidins for the last 24 h. Cells were lysed and periostin protein expression monitored by immunoblotting. The intensity of the immunoreactive bands was analyzed by densitometry using ImageJ software and expressed as a ratio of periostin to housekeeping protein levels to correct for variations in the amount of proteins loaded. The relative levels of proteins were also normalized to TGF-β stimulated cells (value = 100%). Statistically significant differences were calculated by one-way ANOVA followed by Bonferroni’s test (^*^P < 0.05, ^**^P < 0.01, and ^***^P < 0.001 versus stimulated control). Data are representative of three or more independent experiments.

The possibility that anthocyanidins could affect TGF-β-induced periostin expression was next examined. As described in the Methods section, U-87 MG cells were incubated with serum-free medium, then pre-, co- or post-treatment was performed in the presence or absence of 50 μM anthocyanidins (Cy, cyanidin; Dp, delphinidin; Pg, pelargonidin; Pt, petunidin; Mv, malvidin). After 48 h of stimulation with TGF-β, periostin expression levels were evaluated by immunoblotting. Interestingly, all anthocyanidins tested inhibited periostin expression whether used under pre-, co- or post-treatment conditions (Figure 3A–3C), except for co-treatment with Mv and TGF-β (Figure 3B). Dp was found the most potent inhibitor of periostin expression.

### Anthocyanidin effects on the expression of TGF-β-induced periostin

**Figure 4:**
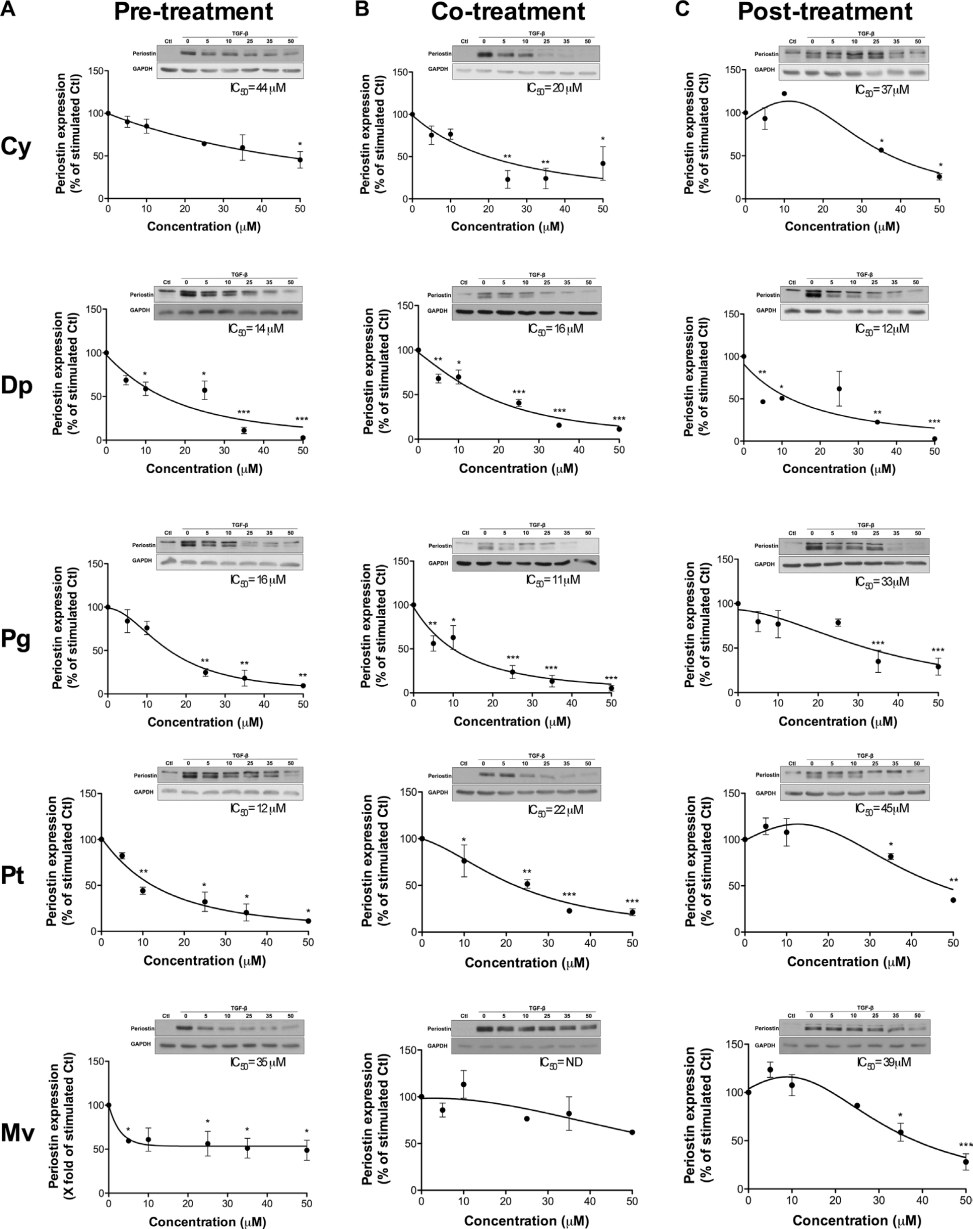
Anthocyanidin effects on the expression of TGF-β-induced periostin U-87 MG cells were treated with various concentrations of each anthocyanidin in serum-free medium (**A**) prior to, (**B**) along with, or (**C**) following addition of 10 ng/mL TGF-β. Cells were lysed and the periostin protein levels assessed by immunoblotting. The immunoreactive band intensities were analyzed by densitometry using ImageJ software and expressed as a ratio of levels of periostin to those of the housekeeping GAPDH protein to correct for variations in the amount of proteins loaded. The relative levels of proteins were also normalized to the TGF-β-stimulated condition (value = 100%). Statistically significant differences were calculated by one-way ANOVA followed by Bonferroni’s test (^*^P < 0.05, ^**^P < 0.01, and ^***^P < 0.001 versus stimulated control). Data are representative of three or more independent experiments.

We next treated U-87 MG cells with various concentrations of anthocyanidins, and determined the half-maximal inhibitory concentration (IC_50_) values on the expression of TGF-β-induced periostin. All five anthocyanidins down-regulated TGF-β-induced periostin expression in pre- (Figure 4A), co- (Figure 4B) and post-treatment (Figure 4C) conditions, except for Mv in the co-treatment condition where we observed a slight non-statistical difference downward trend (Figure 4B). Dp was the most potent inhibitor of TGF-β induced periostin expression with IC_50_ values of 14, 16 and 12 μM for pre-, co- and post-treatment conditions, respectively. Cy and Mv were less efficient with IC_50_ values ranging from 35–44 μM for pre- and post-treatment conditions. Pg and Pt were able to down-regulate TGF-β-induced periostin expression with lower IC_50_ values for pre- (16 and 12 μM, respectively) and co-treatments (11 and 22 μM, respectively) in comparison to post-treatment (33 and 45 μM, respectively). Overall, each anthocyanidin was efficient in down-regulating TGF-β-induced periostin expression whether under pre-, co- or post-treatment conditions.

### Periostin regulates TGF-β-induced U-87 MG cell invasion, cell migration and the Smad2, Akt and Fak signaling pathways

**Figure 5:**
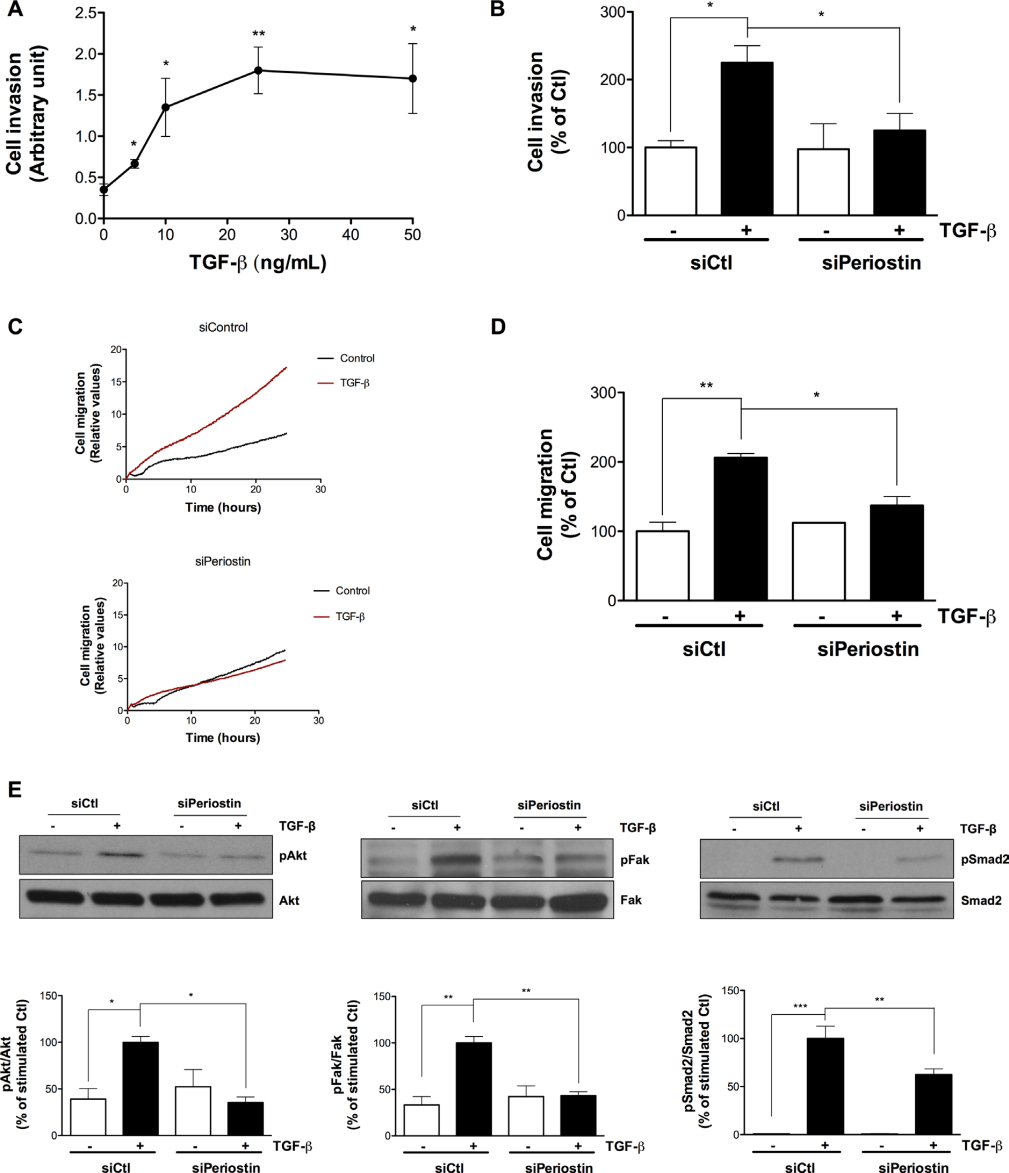
Periostin regulates TGF-β-induced U-87 MG cell invasion, cell migration and Smad2, Akt and Fak signaling pathways U-87 MG cells were (**A**) treated with different concentrations of TGF-β for 48 h, or (B-E) transfected with 2 nM periostin siRNA for 24 h then treated with 10 ng/mL TGF-β for 48 h before (**B**) adhesion onto CIM-Plates coated (or not) with Matrigel or (C, D) adhesion onto CIM-Plates coated with 0.15% gelatin or (E) cell lysis and immunoblotting. Data showing (A, B) TGF-β-induced invasion of U-87 MG cells at 10 h, (**C**) Real-Time migration of TGF-β-induced U-87 MG cells and (**D**) TGF-β-induced migration of U-87 MG cells at 10 h. Values are means ± SEM of three independent experiments performed in quadruplicate. Cell invasion represents the percentage of the ratio of cell index of Matrigel-coated wells to cell index of uncoated wells. Cell migration represents the percentage of the normalized cell index of coated wells. (**E**) Western blot analysis shows levels of phosphorylated proteins. The immunoreactive band intensities were analyzed by densitometry using ImageJ software and expressed in arbitrary units as a ratio of levels of phosphorylated protein to those of the total protein to correct for variation in the amount of loaded proteins. The relative levels of phosphorylated proteins were also normalized to that of TGF-β stimulated controls. Statistically significant differences were calculated by one-way ANOVA followed by Bonferroni’s test (^*^P < 0.05, ^**^P < 0.01, and ^***^P < 0.001 versus stimulated control).

Since processes of cell invasion [5, 18] and cell migration [19] are crucial during EMT, we tested whether periostin is involved in TGF-β-induced U-87 MG invasion and migration. We previously established the concentration of TGF-β to be used in the cell migration assay with the xCELLigence Real-Time Cell Analysis system [20]. Here, we first determined the appropriate concentration of TGF-β to be used for the invasion assay. Prior to the U-87 MG cell invasion or migration assay, TGF-β was added to cells for 48 h and found to trigger invasion in a dose-dependent manner, reaching a plateau between 10–25 ng/mL (Figure 5A). In order to keep the same treatment conditions, TGF-β was used at 10 ng/mL to study periostin’s effect on cell invasion. When periostin was silenced, the TGF-β-induced cell invasion was decreased by 80% (Figure 5B). This confirms that periostin is effectively involved in TGF-β-induced U-87 MG cell invasion. Furthermore, cell migration was decreased in a time-dependent manner when periostin was silenced (Figure 5C) and by ∼ 60% after 10 h of TGF-β-induced migration (Figure 5D). These results confirm the important role of periostin in cell migration. Next, we found that periostin repression reduced the TGF-β-mediated phosphorylation status of Akt and Fak by ∼ 60% for both (Figure 5E), in accordance with previous reports [21–23]. Finally, TGF-β-induced phosphorylation of the Smad2 signaling pathway was lowered by 40% (Figure 5E) when periostin was repressed.

### Anthocyanidins alter the Akt and Fak signaling pathways

**Figure 6:**
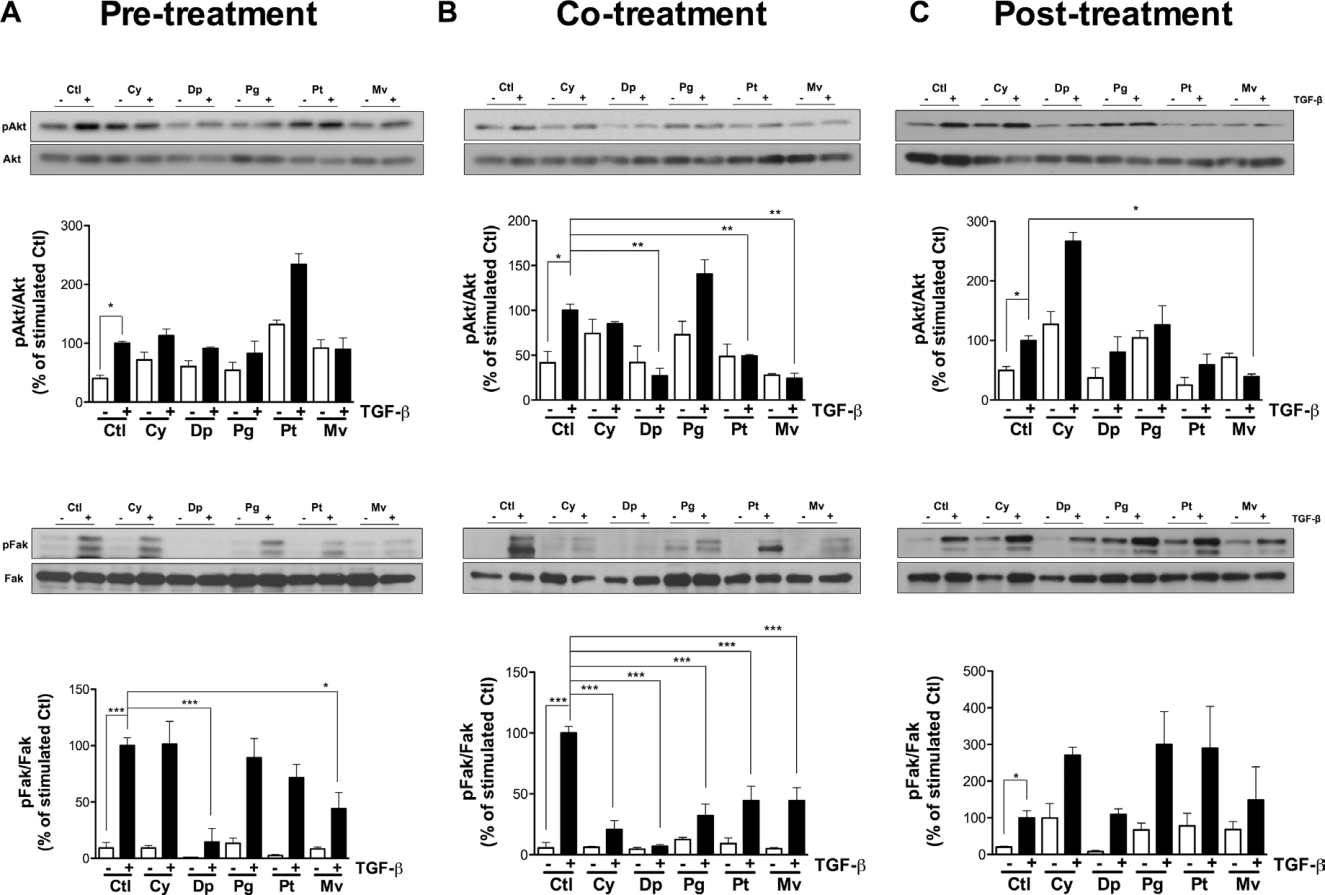
Anthocyanidins alter Akt and Fak signaling pathways U-87 MG cells were incubated in serum-free medium containing (or lacking) 50 μM of the indicated anthocyanidin. Cells were pre-treated with (**A**) anthocyanidins for 24 h, followed by 10 ng/mL TGF-β for 48 h, or (**B**) serum starved for 24 h and co-treated with anthocyanidins and TGF-β for 48 h, or (**C**) serum starved for 24 h followed by the addition of TGF-β for 48 h and post-treated with anthocyanidins for the last 24 h. After treatments, levels of phosphorylated Akt and Fak proteins, along with their individual total protein levels, were monitored by immunoblotting using specific antibodies. The immunoreactive band intensities were analyzed by densitometry using ImageJ software and expressed in arbitrary units as a ratio of levels of phosphorylated protein to those of the total protein to correct for variation in the amount of protein. The relative levels of phosphorylated protein were also normalized to stimulated controls (value = 100%). Statistically significant differences were calculated by one-way ANOVA followed by Bonferroni’s test (^*^P < 0.05, ^**^P < 0.01, and ^***^P < 0.001 versus stimulated controls). Data are representative of three or more independent experiments.

TGF-β is known to trigger cell migration [24, 25] and cell invasion [26] via phosphorylation of Akt and Fak. Consequently, we examined anthocyanidins effects on TGF-β-induced Akt and Fak phosphorylation. U-87 MG cells were treated with 10 ng/mL TGF-β for 48 h in pre-, co- and post-treatment condition in the presence of 50 μM anthocyanidins (Figure 6), and the phosphorylation status of Akt and Fak were examined using immunoblotting. When compared to similarly stimulated controls, Dp and Mv decreased the ratio of pFak/Fak in pre-treatment condition by 80 and 55% respectively (Figure 6A). None of the five anthocyanidins tested was able to down-regulate the ratio of pAkt/Akt in pre-treatment or pFak/Fak in post-treatment conditions. On the other hand, co-treatments with TGF-β and Dp, Pt and Mv or post-treatments with Mv, significantly decreased the pAkt/Akt ratio by more than 50% (Figure 6B and 6C). Interestingly, all five anthocyanidins were able to attenuate phosphorylation of Fak, with inhibition ranging from 50 to 90% under co-treatment conditions (Figure 6B). These results indicate that anthocyanidins are able to affect TGF-β-induced phosphorylation of Akt and Fak in U-87 MG cells.

### Galunisertib inhibits TGF-β-induced EMT markers, Smad2, Akt and Fak signaling pathways

**Figure 7:**
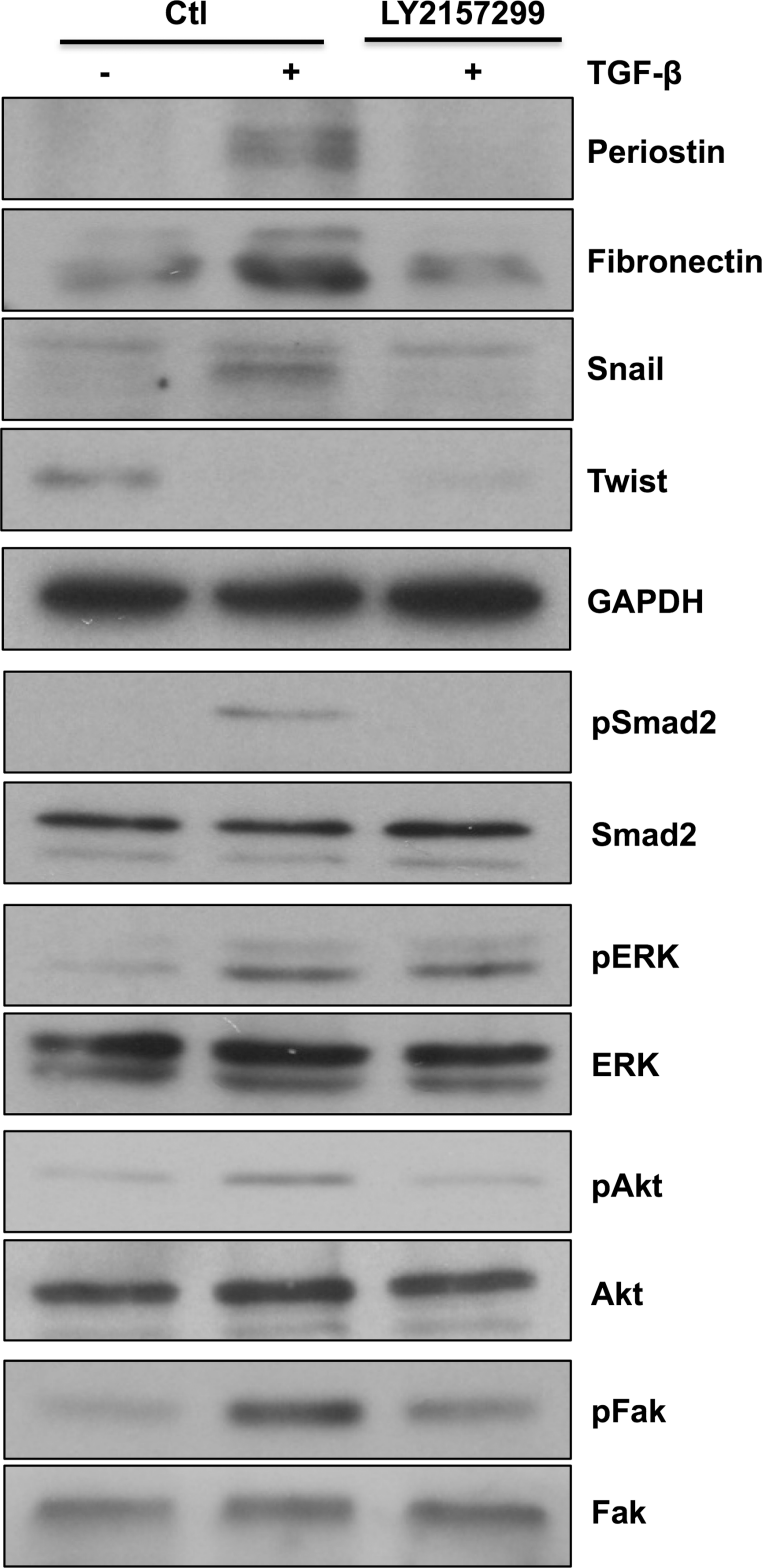
Galunisertib alters TGF-β-induced EMT markers, Smad2, Akt and Fak signaling pathways U-87 MG cells were serum-starved for 24 h, then incubated in serum-free medium containing (or lacking) 10 μM Galunisertib (LY2157299) and 10 ng/mL of TGF-β for 48 h. Cells were lysed and expression levels of EMT markers periostin, fibronectin, Snail and twist, or the phosphorylation status of Smad2, Erk, Akt and Fak proteins were monitored by immunoblotting.

Since TGF-β has an important role in tumorigenesis and contributes to tumor proliferation, invasion and metastasis [27], several approaches have been developed to counteract its signaling function. One of the TGF-β inhibitors, Galunisertib (LY2157299 monohydrate), has antitumor activity exerted, in part, through specific inhibition of Smad2 phosphorylation [28]. In this study, we compared anthocyanidins to Galunisertib with regard to TGF-β-induced EMT. Since we have demonstrated inhibitory effects of anthocyanidins on EMT markers expression [20], we tested whether Galunisertib similarly affected these targets. Galunisertib was shown to be efficient at inhibiting periostin, fibronectin and Snail expression induced by TGF-β (Figure 7). Similar to the anthocyanidins, Galunisertib was unable to reverse Twist down regulation, whereas Smad2 and Akt phosphorylation were completely inhibited. Furthermore, we noticed a down-regulation of Fak phosphorylation when U-87 MG cells were treated with Galunisertib along with TGF-β with no effect on Erk phosphorylation (Figure 7). Taken together, these results show that anthocyanidins, similar to the phase II experimental drug Galunisertib, target several common EMT biomarkers.

## DISCUSSION

We have, in a previous study, validated that the U-87 MG cell line is a suitable model to study EMT since treatment with TGF-β lead these cells to adopt a mesenchymal-like morphology and to express high levels of fibronectin, Snail and slug [20]. Effectively, there is a marked difference either in the stimulation of the marker expression of periostin, Snail and fibronectin or in the down-regulation of Twist by TGF-β in U-87 MG cells as compared to Hs 683, T98G, U-118 MG, U-138 MG and U-251 MG (Supplementary Figure 1). In the present study, we questioned the role of periostin in TGF-β-mediated EMT. We first found that TGF-β effectively induced periostin expression in U87-MG cells, in accordance with studies in PC3 and DU145 prostate cancer cell lines [29]. Here, we also demonstrate for the first time the importance of periostin in the TGF-β-induced EMT in U-87 MG cells as silencing periostin in U-87 MG cells strongly decreased fibronectin and vimentin levels. Thus, periostin is a key signaling intermediate protein in the regulation of EMT-associated mesenchymal markers in U-87 MG cells.

Glioblastoma (GBM) can be difficult to treat due to its highly invasive and infiltrative phenotype [12]. Few anticancer drugs have been evaluated with GBM in clinical trials and, unfortunately to date, no drug can efficiently treat or prevent GBM recurrence [12]. Preventing EMT, which is activated during cancer invasion and metastasis, has thus become a novel strategy for suppression of tumor progression [14]. Periostin is critical for the EMT process in several types of tumor cells [29–34]. Reports have shown that 293T cells expressing ectopic periostin contributed to EMT and metastatic potential by up-regulating vimentin, epidermal growth factor receptor (EGFR) and matrix metalloproteinase-9 (MMP-9) expression [34]. Up-regulation of periostin has been shown to regulate EMT in adamantinomatous craniopharyngioma cells through the Akt signaling pathway [30].

It is well known that modifying lifestyle and dietary habits may reduce the incidence of several cancers. Epidemiological, *in vitro* and *in vivo* studies have shown a decreased incidence of cancer for populations which consume a large variety of fruits, vegetables, nuts, whole grains, fish and olive oil, as is the case for the Mediterranean countries [35, 36]. Such dietary regimen contains many polyphenolic compounds that are known to confer anticancer properties [37]. Among these phytochemicals, anthocyanins demonstrate a wide range of pharmacological effects such as in obesity control, prevention of cardiovascular diseases, anti-inflammatory effect, anti-tumor activity and protective effects against the skin toxicity induced by radiotherapy [15, 38]. Our knowledge regarding the effect of diet-derived molecules on periostin is poor. We sought to investigate the anthocyanidins, aglycone forms of anthocyanins, effects on TGF-β-induced periostin. We demonstrated that, except for co-treatment with Mv, the five anthocyanidins tested significantly decreased TGF-β-induced periostin expression whether in pre-, co- or post-treatment conditions. It is important to note that anthocyanin plasma concentrations can reach the mM range [39, 40], and that anthocyanins and their metabolites are able to cross the blood-brain barrier and enter the brain [41]. Thus, anthocyanidins could be promising pharmacological agents for targeting TGF-β-induced EMT through periostin inhibition.

Cell migration and invasion are key steps in the progression of a tumor leading to a malignant phenotype [11, 42, 43]. It has been reported that periostin expression is correlated with glioma grade and that secreted periostin promoted glioma invasion and adhesion [9]. On the other hand, little is known about the role of periostin in glioma cell migration. Here, we demonstrated the importance of periostin in TGF-β-induced U-87 MG invasion and migration. The transient knockdown of periostin reduced these two processes. Thus, periostin is a key player in U-87 MG cell invasion and migration in part through regulation of the Smad2, Akt and Fak signaling pathways. Indeed, our current study demonstrates an inhibitory effect of Cy, Dp, Pg and Pt on the TGF-β-induced phosphorylation of Smad2 with the greatest effect provided by Dp [20]. Moreover, we found that Akt phosphorylation was decreased by Dp, Pt and Mv in co-treatment, and by Mv only in post-treatment conditions. On the other hand, Fak phosphorylation was reversed by all anthocyanidins in co-treatment and by Dp and Mv in pre-treatment. Overall, Mv was the most potent inhibitor followed by Dp against TGF-β-induced phosphorylation of the Akt and Fak signaling pathways. Based on our own previous study [20], we further confirm that the inhibition of Akt and Fak phosphorylation was due to anthocyanidin treatments and not cytotoxicity.

The mechanisms by which anthocyanidins target TGF-β-induced periostin also deserved further consideration. Here, we demonstrated that anthocyanidins alter several TbRI/TbRII and integrin downstream signaling molecules (i.e. Smad2, Akt and Fak). It has been reported that integrins are involved in the activation of TGF-β in LN-308 and LNT-229 glioblastoma cell lines [44]. Indeed, exposure to av, β3 or β5 neutralizing antibodies, RNA interference-mediated integrin gene silencing or the antiangiogenic agent Cilengitide, results in reduced phosphorylation of Smad2 as well as reduced TGF-β protein and gene levels [44]. Few studies have reported protective roles for anthocyanins in cardiovascular diseases through integrin inhibition [45–47]. Given periostin capacity to interact with integrin, we can safely speculate that anthocyanidins act through integrin to alter TGF-β-induced periostin expression in U-87 MG cells.

The inhibitory potential of anthocyanidins is naturally related to their structures, especially to the presence of free hydroxyl groups on the B ring [48]. In our study, Dp showed a better inhibition of TGF-β-induced periostin expression and Akt/Fak phosphorylation in pre-, co-, and post-treatment conditions followed by Cy, Pg, Pt and Mv. These results are consistent with our previous findings [20] where Dp also showed better inhibition of TGF-β-induced mesenchymal markers and TGF-β-induced Smad2 phosphorylation in pre-, co- and post-treatment followed by Cy, Pg and Pt in U-87 MG cells. However, the Mv effect was not previously assessed. It has been reported that substitution of the hydroxyl groups of the B ring with methoxy groups diminished the antioxidant activity of anthocyanidins [48]. This may explain why Mv, which has 3′ and 5′ -dimethoxyl substituents, showed lower inhibition potency than did the 3′, 4′, 5′-trihydroxylated analogue, Dp.

**Figure 8:**
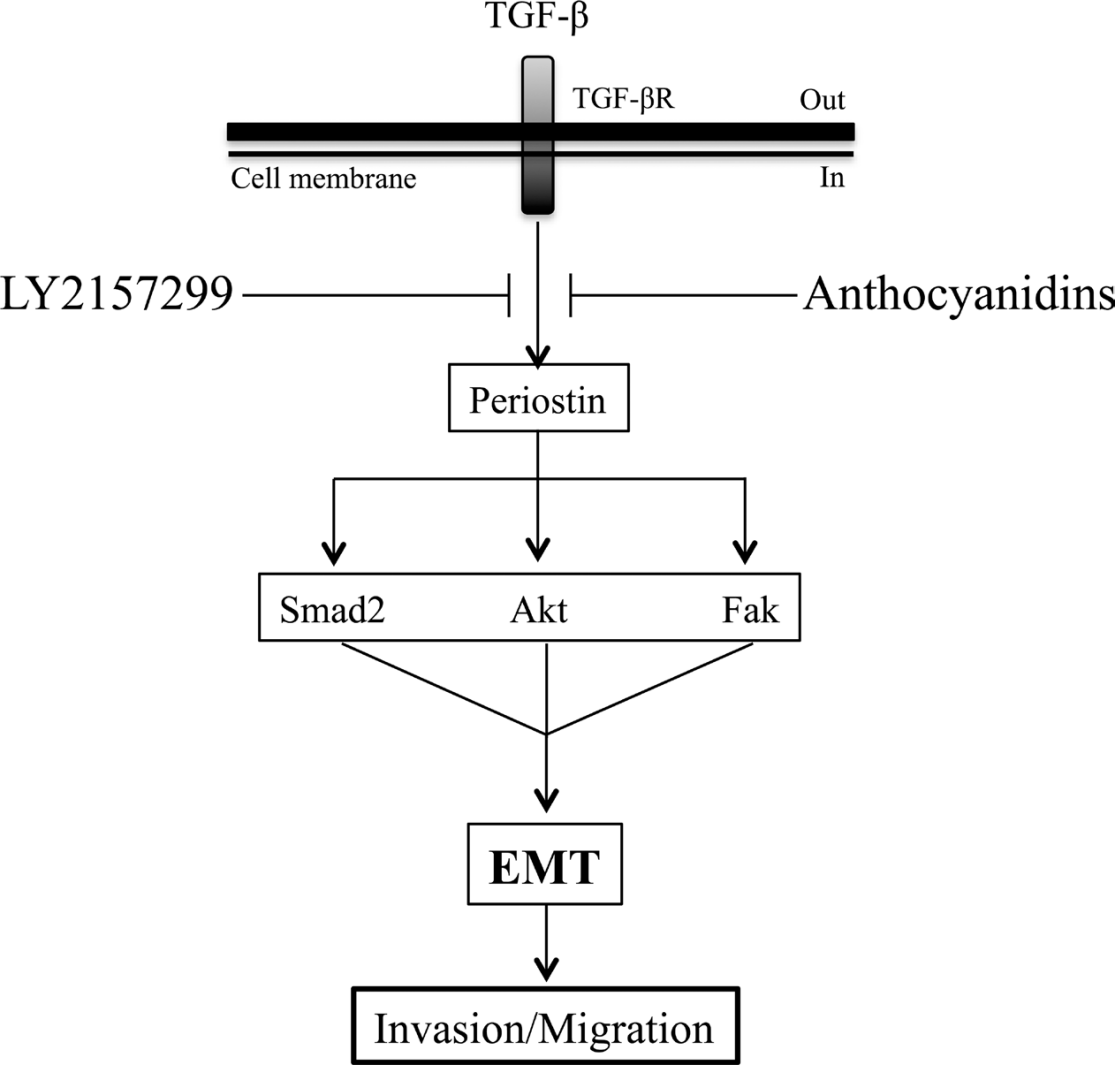
Schematic representation of anthocyanidin and Galunisertib effects on TGF-β-induced EMT in U-87 MG cells TGF-β-induced periostin expression can be suppressed by anthocyanidins and Galunisertib. Smad2, Akt and Fak signaling pathways are activated downstream by periostin upon TGF-β treatment. This is reversed by anthocyanidins or Galinosertib treatments, or by periostin silencing. Anthocyanidins block cancer cell invasion, cell migration and EMT, in part, through periostin downregulation. Based on these evidences, periostin may possibly be used as a therapeutic target for EMT in glioblastoma.

Therapeutic targeting of the TGF-β pathway can slow down tumor progression and metastasis in patients with cancer [49]. Interestingly, several TGF-β pathway inhibitors have reached clinical studies such as, Fresolimumab (monoclonal antibody), Lucanix (vaccine), Galunisertib (small molecule inhibitor), Vigil (vaccine), and Trabedersen (antisense oligonucleotide). Among those inhibitors, Galunisertib (LY2157299), a small molecule developed by Eli Lilly and company, is the most advanced with promising results in phase II clinical trials [17, 50]. However, it has been reported that long-term exposure to Galunisertib caused cardiac toxicities in animal models [28]. In our study, we compared Galunisertib and anthocyanidins by measuring their inhibitory profiles on TGF-β-induced EMT markers and pathways in a glioblastoma cell line. It is known that Galunisertib is being investigated in patients with glioblastoma, pancreatic cancer and hepatocellular carcinoma [28]. We confirmed that Galunisertib was able to inhibit TGF-β-induced Smad2 phosphorylation in our glioblastoma cell model, which is supported by previous studies in hepatocellular carcinoma [51, 52]. Our results further showed that Galunisertib was able to inhibit TGF-β-induced periostin, fibronectin and Snail expression as well as the TGF-β-induced Akt and Fak phosphorylation, again as shown by data in uterine carcinocarcoma cell lines [53]. Furthermore, it has been shown that Akt phosphorylation decreased in an A172 glioblastoma cell line model treated with Galunisertib [16]. Finally, in HT29 colorectal adenocarcinoma and A549 lung carcinoma cell line models, norepinephrine-induced Snail and vimentin expression were inhibited by Galunisertib [54]. However, Galunisertib was unable to reverse twist inhibition or Erk phosphorylation induced by TGF-β (our data). It is interesting to note that the pharmacological action of Galunisertib shares common targets with anthocyanidins, as it is illustrated in Figure 8. Also, we previously demonstrated that anthocyanidins were able to reverse TGF-β-induced EMT in U-87 MG cells by decreasing mesenchymal markers (fibronectin and Snail) and Smad2 phosphorylation, but were unable to reverse Twist and Erk phosphorylation [20]. As Galunisertib is being investigated in monotherapy modalities and in combination with antitumor regimens [28], it may be legitimate to envision testing consumption of anthocyanidins along with or prior to Galunisertib treatment.

Current cancer management which consists of surgery, chemotherapy and radiotherapy presents limitations such as affordability, availability and undesirable side and adverse effects [55]. Thus, the use of natural diet-derived products for cancer prevention has received remarkable attention in recent years [55]. In this study, we have used three different treatment protocols: pre-, co- and post-treatment of anthocyanidins with TGF-β. The pre-treatment condition protocol mimics prevention of TGF-β-induced EMT. On the other hand, co- and post-treatment conditions mimic a TGF-β-induced EMT as would be associated with advanced cancers. We demonstrated that the five anthocyanidins tested were effective in inhibition or in prevention of TGF-β-induced EMT in glioblastoma cells.

Overall, our study highlights periostin as a new target of anthocyanidins. This prometastatic protein can be induced by TGF-β and downregulated by anthocyanidins in U-87 MG cells whether under pre-, co- or post-treatment conditions. Given the operative SMAD2-mediated signaling pathway, high Snail induction, and optimal Twist expression inhibition, we also believe that it is pertinent to the scientific community to highlight U-87MG cells as the best model to study TGF-β-induced EMT. Our study finally demonstrates that blocking periostin expression significantly alters TGF-β-induced EMT and U-87 MG cell invasion and migration. These findings indicate that periostin is a potential therapeutic target for suppressing metastatic progression of glioblastoma cells.

## MATERIALS AND METHODS

### Materials

Anthocyanidin compounds Cy (purity ≥ 96%), Dp, Pg, Mv (purity ≥ 97%) and Pt (purity ≥ 95%) were from Extrasynthese (Lyon, France). Recombinant human TGF-β1 was from R&D Systems (Minneapolis, MN). Electrophoresis reagents were from Bio-Rad (Mississauga, ON). The anti-Twist1 monoclonal antibody was from Santa Cruz Biotechnologies (Dallas, TX). The anti-Periostin antibody was from Abcam (Cambridge, MA). Antibodies against Snail, phospho-Smad2, Smad2, GAPDH (glyceraldehyde-3-phosphate dehydrogenase), vimentin, β-Catenin, phospho-Fak, Fak, phospho-Akt and Akt were from Cell Signaling Technology (Beverly, MA). The anti-fibronectin antibody was from Sigma Aldrich (Oakville, ON). Anti-mouse and anti-rabbit horseradish peroxidase (HRP)-linked secondary antibodies were from Jackson ImmunoResearch Laboratories (West Grove, PA) and enhanced chemiluminescence (ECL) reagents were from Denville Scientific Inc. (Metuchen, NJ). Micro bicinchoninic acid protein assay reagents were from ThermoFisher Scientific (Rockford, IL).

### Cell culture

The human U-87 MG cell line was purchased from American Type Culture Collection (Manassas, VA). Cells were maintained in Eagle’s Minimal Essential Medium (Wisent, 320-036-CL) supplemented with 10% calf serum (HyClone Laboratories, SH30541.03), 1 mM sodium pyruvate (Sigma-Aldrich, P2256), 2 mM L-glutamine, 100 units/mL penicillin and 100 mg/mL streptomycin (Wisent, 450–202-EL), and cultured at 37°C under a humidified 95%–5% (v/v) mixture of air and CO_2_. Cells were treated with vehicle (0.1% ethanol) or with anthocyanidins in the absence of serum prior to (pre-treatment), along with (co-treatment) or following (post-treatment) addition of 10 ng/mL TGF-β.

### Western blot analysis

In order to study the expression of periostin during TGF-β treatment, different concentrations of TGF-β (0–50 ng/mL) were added to the cells, then a time course was carried out by adding 10 ng/mL for 24, 48 and 72 h. The effect of periostin knockdown on EMT markers, as well as the effect of anthocyanidins on periostin expression and on Akt and Fak phosphorylations, was also evaluated by addition of anthocyanidins (0–50 mM) to the cells as described below. U-87 MG cells were pre-treated with anthocyanidins for 24 h, followed by TGF-β for 48 h, or serum starved for 24 h and co-treated with anthocyanidins and TGF-β for 48 h, or serum starved for 24 h followed by the addition of TGF-β for 48 h and then post-treated with anthocyanidins for the last 24 h. Cells were then washed with ice-cold phosphate-buffered saline (PBS) containing 1 mM each of sodium fluoride (NaF) and sodium orthovanadate (Na_3_VO_4_) and incubated in the same medium for 30 min at 4°C. Cells were solubilized in lysis buffer [150 mM NaCl, 10 mM Tris–HCl, pH 7.4, 1 mM EDTA, 1 mM ethyleneglycol-O, O’-bis (2-aminoethyl)-N, N, N’, N’-tetraacetic acid (EGTA), 0.5% (vol/vol) Nonidet P-40 and 1% (vol/vol) Triton X-100]. The resulting lysates were solubilized in Laemmli sample buffer [125 mM Tris-HCl (pH 6.8), 20% glycerol, 4% SDS, 10% β-mercaptoethanol, and 0.00125% bromophenol blue], boiled for 4 min, and separated by sodium dodecyl sulfate-polyacrylamide gel electrophoresis (SDS-PAGE). After electrophoresis, proteins (20 mg) were transferred to polyvinylidene difluoride (PVDF) membranes, which were then blocked for 1 h at room temperature (RT) with 5% non-fat dry milk in Tris-buffered saline/Tween 20 (TBS-T; 147 mM NaCl, 20 mM Tris-HCl, pH 7.5, and 0.1% Tween 20). Membranes were further washed three times in TBS-T and incubated with the primary antibody in TBS-T containing 3% bovine serum albumin (BSA) and 0.03% sodium azide, followed by a 1 h incubation with HRP-conjugated anti-mouse or anti-rabbit antibodies in TBS-T containing 5% non-fat dry milk. Immunoreactive material was visualized using an ECL detection system. The immunoreactive bands were quantified using ImageJ software (NIH).

### Immunofluorescence

U-87 MG cells were seeded on cover slips and treated with vehicle (0.1% ethanol) or 10 ng/mL TGF-β for 48 h. Cells were washed twice with ice-cold PBS, fixed in 3.7% formaldehyde for 20 min at RT and permeabilized with 0.1% Triton X-100 for 5 min. Non-specific binding was blocked with 1% BSA containing 0.03% sodium azide in PBS overnight at 4°C. After 3 washes with ice-cold PBS for 15 min, cells were then incubated with the primary anti-periostin antibody (1:400) for 1 h at RT, washed twice with cold PBS and incubated with Alexa Fluor-568-conjugated anti-rabbit IgG (Invitrogen, CA) at 1:200 for 1 h at RT. The cell nuclei were visualized using 1 mg/mL 4′, 6-diamidino-2-phenylindole (DAPI) staining for 5 min at RT. Slides were then dried, mounted with the ProLong Gold antifade reagent (ThermoFisher Scientific, Ottawa, ON) and fluorescence examined by confocal microscopy.

### siRNA transfections

Periostin siRNA (Sense: 5′-GCGCCUCCUUAAAUUAAAUUAAUUTT-3′; Antisense: 5′-AAUUAAUUUAAGGAGGCGCTG-3′) was from Qiagen (Valencia, CA). Prior to transient transfections with both types of siRNAs, U-87 MG cells were plated into 6-well plates and grown until reaching 70% confluence. U-87 MG cells were transfected for 24 h with 2 nM of either siRNA using Lipofectamine 2000 reagent as described in the manufacturer’s instructions (ThermoFisher Scientific, Ottawa, ON). After transfection, cells were treated with 10 ng/mL TGF-β for 48 h.

### Quantitative PCR analysis

After cell transfection, total RNA was isolated using TRIzol reagent (Life Technologies, Gaithersburg, MD) and cDNA was synthesized using a high capacity cDNA reverse transcription kit (Applied Biosystems, Foster City, CA) according to the manufacturer’s instructions. Quantitative PCR was performed using SsoFast EvaGreen Supermix (Bio-Rad, Hercules, CA). DNA amplification was carried out using a CFX Connect Real-Time System (Bio-Rad) and product detection was performed by measuring binding of the fluorescent dye EvaGreen to double stranded DNA. The QuantiTect primer sets for GAPDH (Hs_GAPDH_2_SG QT01192646) and β-actin (Hs_Actb_2_SGQT01680476) were from Qiagen whereas those for periostin (322445G05 (hPerioS) and 322445G06 (hPerioAS)) were from Invitrogen. The relative quantities of target gene mRNA were compared against two internal controls, GAPDH and β-actin mRNA, and measured by following a ΔCT method employing an amplification plot (fluorescence signal vs. cycle number). The difference (ΔC_T_) between the mean values in triplicate samples of the target gene and those of GAPDH and β-actin mRNAs were calculated by CFX Manager software version 2.1 (Bio-Rad). The relative quantified value (RQV) was expressed as 2^− ΔC^T.

### Real-time cell invasion assay

Invasion assays were carried out using the Real-Time Cell Analyser (RTCA) Dual-Plate (DP) Instrument and the xCELLigence system (Roche Diagnostics, QC) following the supplier’s instructions. The optimal concentration of TGF-β for invasion assay was first determined. U-87 MG cells were transfected with 2 nM siRNAs (Control and periostin) ± TGF-β as described above. After transfection, the upper chamber of a CIM-plate 16 (Roche Diagnostics) was coated with growth factor-reduced Matrigel (BD Biosciences, Bedford, MA) and allowed to polymerize at 37°C for 4 h. The lower chamber was filled with serum-free medium and the upper chamber of each well was filled with 20,000 cells. After 30 min of adhesion, cell invasion was monitored every 15 min for 24 h under a humidified atmosphere containing 5% CO_2_. The impedance value was measured using the RTCA DP Instrument and expressed as an arbitrary unit called the Cell Index. Each experiment was performed in quadruplicate wells. The results were represented by cell invasion, which represents a ratio of cell index of Matrigel-coated wells to cell index of uncoated wells at a given time point.

### Real-time cell migration assay

Experiments were carried out using the Real-Time Cell Analyser (RTCA) Dual-Plate (DP) Instrument and the xCELLigence system (Roche Diagnostics, QC), following the instructions of the supplier. U-87 MG cells were transfected with 2 nM siRNAs (Control and periostin) ± TGF-β as described above. After transfection, 250 000 cells per well were seeded in a CIM-plate 16 (Roche Diagnostics), and incubated at 37°C under a humidified atmosphere containing 5% CO_2_ for 24 h. Prior to cell seeding, the underside of each well in the upper chamber was coated with 0.15% gelatin in PBS and incubated for 1 h at 37°C. The lower chamber was filled with serum-free medium. The upper chamber of each well was filled with 250000 cells. After 30 min of adhesion, cell migration was monitored every 5 min for 24 h. The impedance value was measured by the RTCA DP Instrument and expressed as an arbitrary unit called the Cell Index. Each experiment was performed in quadruplicate wells.

### Statistical analysis

Statistical analyses were generally performed using one-way ANOVA with a post hoc test Bonferroni’s test. Student’s unpaired *t* test was used to determine the statistical significance between the control and the stimulated control for periostin gene expression. Differences with *P* < 0.05 were considered significant. All statistical analyses and graphs were performed using the GraphPad Prism software version 5.0b (San Diego, CA).

## SUPPLEMENTARY MATERIALS FIGURE



## Abbreviations

CNS: central nervous system
Cy: cyanidin
Dp: delphinidin
EMT: epithelial mesenchymal transition
GBM: glioblastoma multiforme
Mv: malvidin
Pg: pelargonidin
Pt: petunidin
TGF-β: transforming growth factor β
