# Supplementary material for: Periostin, a signal transduction intermediate in TGF-β-induced EMT in U-87MG human glioblastoma cells, and its inhibition by anthocyanidins
Source: Oncotarget. 2018 Apr 24;9(31):22023–37. doi: 10.18632/oncotarget.25153 (PMC5955165; doi:10.18632/oncotarget.25153)
Supplement: Supplementary file 1 [file oncotarget-09-22023-s001.pdf]

Periostin, a signal transduction intermediate in TGF-β-induced EMT in U-87MG human glioblastoma cells, and its inhibition by anthocyanidins

SUPPLEMENTARY MATERIALS

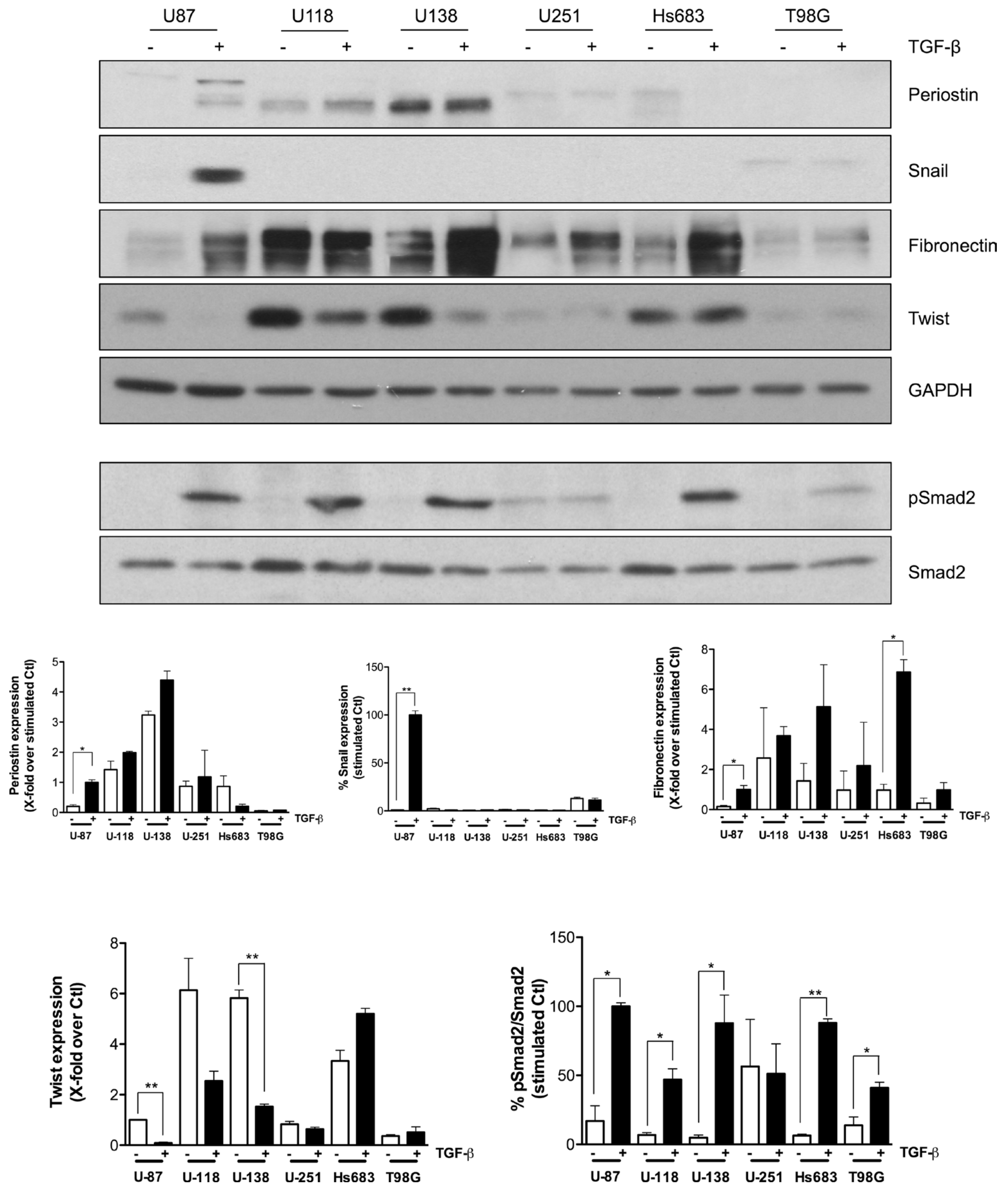

Supplementary Figure 1: TGF-β-induced EMT markers and Smad2 phosphorylation in different glioblastoma cell lines. U-87 MG, U118, U138, U251, Hs683 and T98G cells were incubated in serum-free medium containing (or lacking) 10 ng/mL of TGF-β for 48 h. Cells were lysed and expression levels of EMT markers periostin, fibronectin, Snail and Twist or the phosphorylation status of Smad2, were monitored by immunoblotting. The immunoreactive band intensities were analyzed by densitometry using ImageJ software. Statistically significant differences were calculated by one-way ANOVA followed by Bonferroni's test ($^{*}P < 0.05$, $^{**}P < 0.01$, and $^{***}P < 0.001$ versus stimulated controls). Data are representative of three or more independent experiments.
